# Regulation of Cardiac Expression of the Diabetic Marker MicroRNA miR-29

**DOI:** 10.1371/journal.pone.0103284

**Published:** 2014-07-25

**Authors:** Nicholas Arnold, Purushotham Reddy Koppula, Rukhsana Gul, Christian Luck, Lakshmi Pulakat

**Affiliations:** 1 Department of Medicine, University of Missouri, Columbia, Missouri, United States of America; 2 Department of Nutrition and Exercise Physiology, University of Missouri, Columbia, Missouri, United States of America; 3 Harry S Truman Memorial Veterans Affairs Hospital, Columbia, Missouri, United States of America; 4 Obesity Research Center, College of Medicine, King Saud University, Riyadh, Saudi Arabia; University of Nebraska Medical Center, United States of America

## Abstract

Diabetes mellitus (DM) is an independent risk factor for heart disease and its underlying mechanisms are unclear. Increased expression of diabetic marker miR-29 family miRNAs (miR-29a, b and c) that suppress the pro-survival protein Myeloid Cell Leukemia 1(MCL-1) is reported in pancreatic β-cells in Type 1 DM. Whether an up-regulation of miR-29 family miRNAs and suppression of MCL-1 (dysregulation of miR-29-MCL-1 axis) occurs in diabetic heart is not known. This study tested the hypothesis that insulin regulates cardiac miR-29-MCL-1 axis and its dysregulation correlates with DM progression. *In vitro* studies with mouse cardiomyocyte HL-1 cells showed that insulin suppressed the expression of miR-29a, b and c and increased MCL-1 mRNA. Conversely, Rapamycin (Rap), a drug implicated in the new onset DM, increased the expression of miR-29a, b and c and suppressed MCL-1 and this effect was reversed by transfection with miR-29 inhibitors. Rap inhibited mammalian target of rapamycin complex 1 (mTORC1) signaling in HL-1 cells. Moreover, inhibition of either mTORC1 substrate S6K1 by PF-4708671, or eIF4E-induced translation by 4E1RCat suppressed MCL-1. We used Zucker diabetic fatty (ZDF) rat, a rodent model for DM, to test whether dysregulation of cardiac miR-29-MCL-1 axis correlates with DM progression. 11-week old ZDF rats exhibited significantly increased body weight, plasma glucose, insulin, cholesterol, triglycerides, body fat, heart weight, and decreased lean muscle mass compared to age-matched lean rats. Rap treatment (1.2 mg/kg/day, from 9-weeks to 15-weeks) significantly reduced plasma insulin, body weight and heart weight, and severely dysregulated cardiac miR-29-MCL1 axis in ZDF rats. Importantly, dysregulation of cardiac miR-29-MCL-1 axis in ZDF rat heart correlated with cardiac structural damage (disorganization or loss of myofibril bundles). We conclude that insulin and mTORC1 regulate cardiac miR-29-MCL-1 axis and its dysregulation caused by reduced insulin and mTORC1 inhibition increases the vulnerability of a diabetic heart to structural damage.

## Introduction

Several epidemiological studies including the Framingham Study, UK Prospective Diabetes Study (UKPDS), Cardiovascular Health Study, and the Euro Heart Failure Surveys provide strong evidence for the fact that diabetes mellitus (DM) is an independent predictor for heart disease [1]–[4]. The fact that the adults with diabetes have heart disease death rates about 2–4 times higher than adults without diabetes strongly suggests that the compensated heart in DM is very vulnerable to sudden malfunction resulting in death. In addition to the well-studied left ventricular (LV) dysfunction in DM, recent studies have highlighted the involvement of right ventricular (RV) dysfunction in diabetic heart disease [5], [6]. However, mechanisms underlying diabetic cardiomyopathy are still elusive. Identifying DM-specific molecular changes that increase the vulnerability of cardiac myofibrils to structural damage is of high utility in developing new therapeutics and regimens to control heart disease in diabetic individuals.

**Figure 1:**
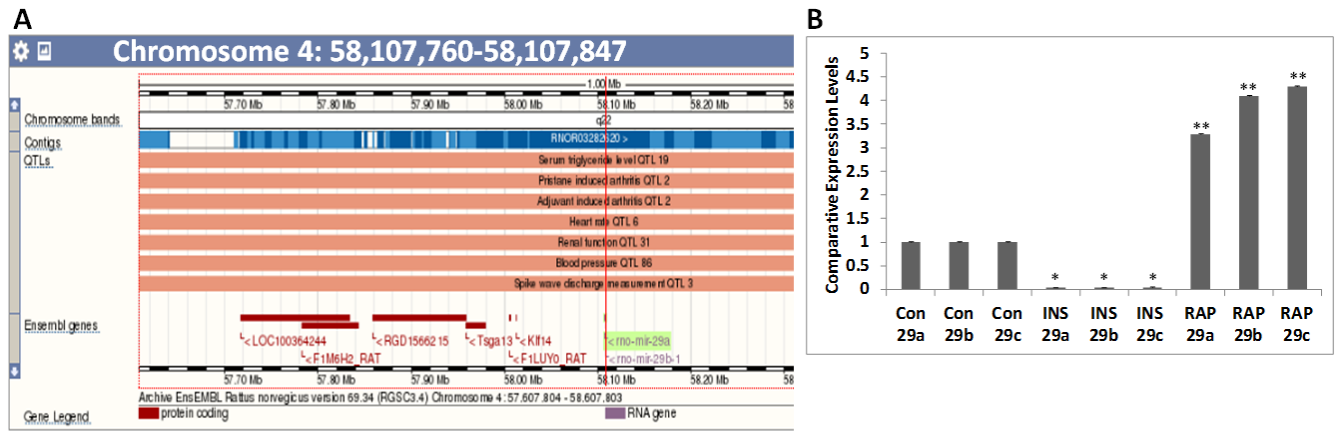
miR-29 family miRNA expression pattern. A) The miR-29a/b cluster is associated with cardiovascular diseases. QTLs associated with the rat (rno)-miR-29 a/b cluster located on chromosome 4: 58,107,760-58,107,847 are shown (Taken from Rat RGSC3.4. http://oct2012.archive.ensembl.org/Rattus_norvegicus/Location/View?g=ENSRNOG00000035458;r=4:58136357-58136365;t=ENSRNOT00000053581). B) Expression of miR-29 family miRNAs (miR-29a, b and c) in mouse cardiomyocyte HL-1 cells is suppressed by treatment with INS (100 nM; 12 h) and up-regulated by treatment with Rap (10 nM; 12 h). Comparative expression levels (RQ values) are expressed relative to untreated (Con) HL-1 cells. Treatments were performed in quadruplicates and qRT-PCR per each biological sample was performed in triplicates. Values are means ± SEM. * p<0.05 for Con vs. INS and ** p<0.05 con vs. RAP for miR-29 a, b, and c.

In this context, the diabetic marker microRNA miR-29 family that plays a role in increasing cell death is particularly noteworthy. The miR-29 family consists of miR-29 a, b (b1 and b2) and c that are located on two different chromosomes (chromosomes 4 and 13 in rat, 1 and 6 in mouse and 1and 7 in human) [7]. Quantitative trait loci (QTLs) associated with rat miR-29a and b highlight potential involvement of miR-29a and b in cardiovascular diseases (Fig. 1A). miR-29a was identified as one of the miRs that was up-regulated in the serum of children with Type 1 DM (T1DM) [8]. In diabetic mice, an increase in miR-29c was associated with podocyte cell death that underlies diabetic nephropathy. Additionally, knock-down of miR-29c suppressed high glucose induced apoptosis of podocytes and improved kidney function [9]. Increase in miR-29b leads to the development of aortic aneurisms [10]. Suppression of miR-29 by anti-miR-29 oligomers protects against myocardial ischemia-reperfusion injury, abdominal aortic aneurism and diabetic nephropathy [9]–[13]. miR-29 is also one of the several miRNAs associated with inflammatory microvesicles [14]. In non-obese diabetic (NOD) mice, up-regulation of miR-29a, b and c caused pancreatic β-cell death via suppression of the myeloid cell leukemia 1 (MCL-1) gene, an essential member of the pro-survival BCL-2 family genes, and marked the first stage of type 1 DM (T1DM) [15]. Thus, the miR-29-MCL-1 axis is a major contributor to pancreatic dysfunction and T1DM.

The role of the miR-29-MCL-1 axis in the progression of DM-associated heart disease is not known. Recent studies have highlighted the importance of MCL-1 in preventing heart failure [16], [17]. It was reported that deletion of *Mcl-1* gene leads to cardiomyocyte disorganization, fibrosis, inflammation, and lethal heart failure. These studies used inducible, cardiomyocyte-specific *Mcl-1* knockout mice [16], [17]. Interestingly, these studies showed that MCL-1-deficient hearts did not have increased apoptosis of cardiomyocytes. Instead, MCL-1 exerted cardiac protection because it was essential for mitochondrial homeostasis and induction of autophagy in response to cardiac stress [16], [17]. Therefore, preserving MCL-1 is essential to maintain cardiac myofibril structure and organization [16], [17]. These observations raised concerns about the potential cardiotoxicity for chemotherapeutics that target MCL-1 [16], [17]. Moreover, in conditions of disease such as DM, how the expression of cardiac MCL-1 is regulated is not known.

Several studies have shown that rapamycin (Rap), a commonly used drug in cancer therapy and for the prevention of restenosis, also causes insulin resistance and development of DM [18]–[24]. Rap promotes DM by inhibiting pancreatic β-cell proliferation and β-cell adaptation to hyperglycemia. Additionally, new onset DM is associated with sirolimus (Rap) therapy used for organ transplants and cancer. Therefore, we posited that Rap treatment could be used as a method to promote rapid progression of DM in a rat model that is genetically prone to develop DM.

Since miR-29 suppresses MCL-1 and the miR-29 family miRNAs are elevated in DM we hypothesized that one of the mechanisms by which DM promotes heart disease is by causing dysregulation of the miR-29-MCL-1 axis and suppressing MCL-1 levels in cardiomyocytes that can lead to cardiomyocyte disorganization. Moreover, since DM is associated with either insulin deficiency or a lack of insulin signaling, we also posited that insulin would suppress the expression of the miR-29 family in cardiomyocytes and up-regulate MCL-1 expression. This study was undertaken to investigate whether insulin regulated the miR-29-MCL-1 axis in cardiomyocytes and if conditions that lead to progressive loss of insulin promote dysregulation of cardiac miR-29-MCL-1 axis and disorganization of cardiomyocytes. Since insulin is an activator of the nutrient sensor kinase mammalian target of rapamycin complex 1 (mTORC1), we further posited that mTORC1-signaling mediates insulin's effects on miR-29-MCL-1 axis. To evaluate the effect of insulin and mTORC1 on miR-29-MCL-1 axis in cardiomyocytes, we used mouse atrial cardiomyocyte cell line HL-1 [25] and tested the effects of Rap treatment on miR-29-MCL-1 axis regulation. To determine how DM progression (natural or advanced by Rap treatment) caused dysregulation of cardiac miR-29-MCL-1 axis and promoted cardiomyocyte disorganization, we used male ZDF rats, a well-established rodent model for advanced DM [5], [6], [26], [27] and evaluated the correlation between regulation of miR-29-MCL-1 axis and disorganization of myofibril bundles in cardiac right ventricle.

## Methods

### Cell culture and treatments

The cardiac muscle cell line HL-1 (a generous gift from Dr. William Claycomb, - Louisiana State University Medical Center) is an atrial cardiomyocyte tumor lineage originally derived from female AT-1 mouse [25]. Cells were cultured in a 37°C incubator in the presence of 5% CO_2_ in complete Claycomb medium using flasks pre-coated with 12.5 µg/ml bovine fibronectin (Sigma) in 0.02% gelatin solution (Sigma, St Louis, MO) as described previously [25], [28]. Confluent cells were washed with 1X phosphate buffered saline (PBS), and incubated in serum-free Claycomb medium prior to treatments with bovine insulin (100 nM:12 hr), rapamycin (10 nM:12 hr), p70S6K1 inhibitor PF-4708671(2 µM;12 hr), or 4E1RCat, an inhibitor of cap-dependent translation (5 µM;12 hr). Insulin was purchased from Sigma-Aldrich Inc., rapamycin from Cell signaling Technology, and PF-4708671 and 4E1RCat were from Tocris Bioscience. At the end of treatments with different agents, medium was removed, cells were rapidly cooled with ice-cold PBS, collected using cell scrapers followed by centrifugation at 3500 rpms for 5 minutes at 4°C, and cell pellets were flash frozen using liquid nitrogen. Cell pellets were stored in a -80°C ultra-freezer until further processing. All treatments were performed at least in triplicate.

### Animals, Rap treatment, and Body composition

All animal procedures used in this study were approved prior to the beginning of these studies by the Harry S. Truman Veterans Memorial Hospital (HSTVMH) Subcommittee for Animal Safety and University of Missouri IACUC. All animals were cared for in accordance with the Guidelines for the Care and Use of Laboratory Animals (National Institutes of Health publication 85-23). Nine-week old male ZL and ZDF rats (24 animals) purchased from Charles River Laboratories were used in this study. Animals were housed at the HSTVMH animal housing facility under standard laboratory conditions (room temperature: 21–22°C; light and dark cycles: 12 h). Rats were maintained on ad libitum food and water. Rapamycin pellets designed to deliver Rap at a concentration of 1.2 mg/kg/day for 21 days (from Innovative Research of America, Inc, Sarasota, FL) or placebo pellets were placed surgically under the skin behind the shoulder blades under brief isoflurane anesthesia and this procedure was repeated to achieve a 6-week treatment. Body composition in ZL and ZDF rats was determined using the EchoMRI 4in1/1100. EchoMRI is the preferred non-invasive method to measure body composition because it is a rapid measurement that can be performed on live, un-anaesthetized animals [29]. The EchoMRI 4in1/1100, is a QMR system that measures lean mass, fat mass, total water, and free water. The rats were placed in an adjustable plastic cylinder to restrict movement. The cylinder (2.75 inches in diameter) has openings on either end to allow the animals to breathe freely. The cylinder was inserted into the EchoMRI for a reading that lasted for 85 seconds.

### Blood and tissue collection, plasma analysis, histopathology

Animals were fasted for 6 hours before blood collection. Blood was collected biweekly from the saphenous vein. Blood was also collected by cardiac puncture at the time of sacrifice according to IACUC approved procedure. Rats were acclimated for two weeks to reduce stress before starting blood draw. The back of the chosen leg was shaved to make the saphenous vein visible. A compression point at the base of the leg was used to make the saphenous vein bulge out. A 20 G needle was used to puncture the vein to collect the blood. Prior to euthanasia, rats were given intraperitoneal injection with 50 mg/kg of sodium pentobarbital to properly anesthetize them. Animals were euthanized by opening of the chest cavity, removal of 6–10 mls of blood from the heart, followed by removal of the heart. Plasma analysis was performed by Comparative Clinical Pathology Services at Columbia. Plasma levels of cholesterol and triglycerides were measured using commercially available assays (Beckman-Coulter, Brea, CA) on an automated clinical chemistry instrument (AU680, Beckman-Coulter, Brea, CA). Glucose and insulin were measured by an automated hexokinase G-6-PDH assay and an ELISA kit specific for rat insulin, respectively.

Heart tissue from right ventricle (RV) was used in this study for RNA, protein and histopathology analysis. Tissues for RNA isolation and protein analysis were rapidly flash frozen in liquid nitrogen in aluminum foil packets that were pre-cooled on dry-ice. Tissues were fixed in 10% neutral buffered formalin (NBF), embedded into paraffin blocks, sections were cut at 4 µm thickness, and were stained with haematoxylin and eosin (H&E), Masson's Trichrome Stain (MTS), and anti-α-actin antibody at Research Animal Diagnostic Laboratory (RADIL), Columbia, Missouri. The stained sections were scanned using the Aperio CS Slide Scanner by WSI Analytics Lab, Department of Pathology and Anatomical Sciences, University of Missouri, Columbia.

### RNA isolation and quantitative real-time RT-PCR

Isolation of mRNA and miRNA from frozen HL-1 cell pellets and RV tissues was performed using mirVana miRNA isolation kit (Ambion) following the manufacturer's protocol. The mRNA and miRNA were quantified using NanoDrop (Thermo Scientific) and stored at −80°C until further processing. c-DNA synthesis for mRNA and miRNA was carried out using Omniscript RT kit from Promega (Madison, WI) and Taqman MicroRNA Reverse Transcription Kit (Applied Biosystems Life Technologies) respectively. Taqman microRNA assay primers for miR-29a, b and c and snRNA (Taqman microRNA Assays) and mouse and rat MCL-1 and 18S RNA primers (Gene Expression Assays) from Applied Biosystems Life Technologies were used in these experiments. qRT-PCR was carried out using cDNA generated from these RNA samples as templates. Experiments were performed in triplicates for each biological sample with Taqman Fast Universal PCR Master Mix 2X, (Applied Biosystems Life Technologies). snRNA and 18s RNA were used as internal controls for miRNA and mRNA respectively. qRT-PCR was performed using the Applied biosystems 7500 Fast PCR system. Relative quantification (RQ) values were obtained by determining ΔCt values followed by determining ΔΔCt values and then RQ values via the equation 2^(−ΔΔCt)^.

### Immunoblotting

Frozen HL-1 cell pellets from various treatments were lysed using ice-cold nonidet lysis buffer [30] supplemented with okadaic acid (0.1 µM) and Na_3_VO_4_ (0.25 mM) to prevent Serine/Threonine and Tyrosine phosphatases. Cell debris was removed by centrifugation and protein in the supernatant was estimated by BCA method (Pierce BCA protein assay kit). Samples were normalized, and lysates corresponding to 60 µg of protein were subjected to SDS-PAGE analysis. Separated proteins were transferred to PVDF membrane (Millipore) by Western blotting. After blocking with 5% BSA, PVDF membranes were probed with primary antibodies for S6K1, phospho-S6K1(pS6K1: pThr^389^), RPS6, phospho-RPS6(pRPS6: pSer^235/236^), 4E-BP, phospho-4E-BP(p4E-BP: pThr^37/^Thr^46^) and MCL-1 in 5% BSA in TBST overnight (1∶1000 dilution of each antibody; all antibodies from Cell Signaling Technology). Blots were then washed with TBST and incubated with horseradish peroxidase-conjugated secondary antibodies (1∶30,000 dilution of each antibody) for 1 hr at room temperature. Binding of the antibodies was detected by Chemiluminescence (Supersignal west femto maximum sensitivity substrate kit; Thermo Scientific), and images were captured using a Bio-Rad ChemiDoc XRS image-analysis system. All experiments were done at least in triplicates. Quantitation of phosphorylated protein band density, normalized to the density of total protein for each sample, was performed using Quantity One software (Bio-Rad Laboratories Inc. Berkeley, CA). Data are reported as the normalized protein band density in arbitrary units.

### Immunofluorescence

Hl-1 cells were grown on cover slips pre-coated with 12.5 µg/ml bovine fibronectin in 0.02% gelatin solution. Transfection was performed using siPORT Amine (Applied Biosystems) according to manufacturer's protocol. 20 nM of miR-29 inhibitor cocktail (mirVana miRNA inhibitors for miR-29a, b and c) or 20 nM Allstars negative siRNA (Qiagen) was used for transfection. After 8 hours, cells were subjected to Rap treatment (10 nM) overnight. Coverslips were washed with PBS, fixed with 4% paraformaldehyde for 20 min at room temperature, permeabilized with 1% Triton-X, washed with PBS-T (1 mL Tween-20/L), and blocked with background sniper (Biocare Medical). Anti-MCL-1 antibody (Cell Signaling Technology) (1∶50 dilution) was added in fluorescent AB diluent (Biocare Medical) and incubated overnight at 4°C. After repeated washing with PBS-T, coverslips were incubated with Alexa Fluor 488 goat anti-rabbit (Invitrogen Inc.) (1∶200 dilution) for 1 hr at room temperature. After washing with PBS-T, coverslips were mounted on slides with Fluoroshield with 4′,6-diamidino-2-phenylindole (DAPI) (Sigma-Aldrich) and visualized using an Olympus IX51 microscope with a UC50 digital camera using cellSense software (Olympus, Center Valley, PA) at equal exposure times. Imaging was done at 60× magnification using oil immersion.

### Determination of cardiomyofibril disarray

Cardiomyofibril disarray was quantified based on a previously published method [31]. We prepared 2 noncontiguous sections per heart (N = 12 for heart tissues). We measured myocyte disarray by semiautomated planimetry of 48 randomly selected regions within the heart sections. Each region contained 648 sections each at 10× magnification. Cardiomyocyte disarray was scored for every section composing the region and the percentage of disarray within regions was then calculated.

### Statistics

Statistical analysis was performed using the SPSS 20 software package. Results were expressed as mean ± SEM (standard error of mean). Differences among groups were tested by using One-Way ANOVA followed up with Tukey's test or t-test, as appropriate, and two-tailed p values are reported. A *p*-value of 0.05 was considered statistically significant.

## Results

### Regulation of diabetic marker miR-29 by insulin and rapamycin in mouse cardiomyocytes

Mouse atrial cardiomyocyte HL-1 cells are widely used for *in vitro* studies to assess the effects of different hormones and drugs on cardiomyocyte signaling. Since increased expression of different members of miR-29 family is associated with DM, we tested the effects of insulin that attenuates the progression of DM, and rapamycin (Rap) that promotes the progression of DM, on the expression of miR-29 family miRNAs in HL-1 cells. Insulin treatment strongly suppressed miR-29a, b and c in cardiomyocytes whereas Rap treatment significantly enhanced expression levels of all three miR-29 family members in HL-1 cardiomyocytes (Fig. 1B). To our knowledge this is the first report that shows insulin is a regulator of miR-29 family miRNAs.

### Regulation of miR-29 target MCL-1 by insulin and rapamycin in mouse cardiomyocytes

**Figure 2:**
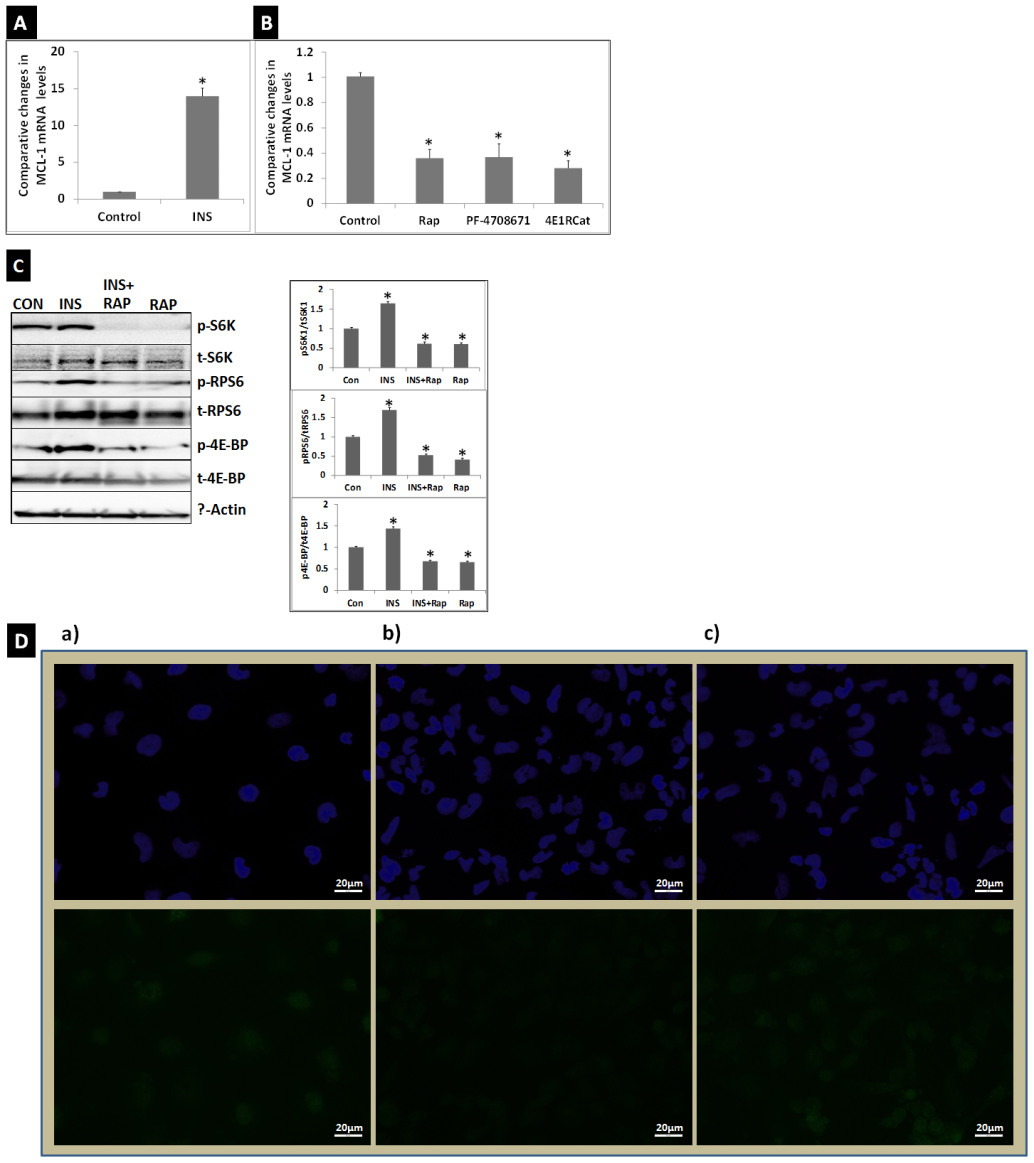
INS and mTORC1 regulate cardioprotective MCL-1 mRNA expression in HL-1 cardiomyocytes. A). MCL-1 expression is up-regulated by treatment with INS (100 nM; 12 h) as determined by qRT-PCR. B) MCL-1 is suppressed by inhibitors of signaling induced by mTORC1 and its substrates p70 s6K1 and 4E-BP. qRT-PCR data showed that MCL1 mRNA levels in HL-1 cardiomyocytes were significantly suppressed in response to treatment with Rap (mTORC1 inhibitor; 10 nM:12 hrs), or PF470867 (p70 s6K1specific inhibitor; 20 nM:12 hrs) or 4E1RCat (a novel suppressor of eIF4E-induced translation; 5 µM; 12 hrs). Treatments were performed at least four times for INS and each of the mTORC1 inhibitors. qRT-PCR for each biological sample was performed in triplicates. Comparative expression levels (RQ values) are expressed relative to untreated (Con) HL-1 cells. Values are means ± SEM. *p<0.01 for Con vs. INS, and Con vs. Rap, PF4708671 or 4E1RCat. C). Autoradiograms show in HL-1 cardiomyocytes levels of pThr^389^S6k1 (pS6K1), pSer^235/236^RPS6 (pRSP6), and pThr^37^/ Thr^46^4E-BP (p4E-BP) were elevated in response to INS(100 n M, 12 hr) treatment and were suppressed in response to Rap (10 nM, 12 Hr with or without INS). Graphs show results of densitometric analysis of the intensity of phosphorylated protein bands after adjusting to the intensity of total protein bands (tS6K1, tRPS6, t4E-BP) and to the intensity of the β-actin that was used as an internal control. N = 4 *p<0.01 for Con vs. INS, INS vs. INS+Rap, and INS vs. Rap. Western blotting also showed suppression of MCL-1 via Rap treatment in HL-1 cardiomyocytes (N = 4, *p<0.0002). There was no significant difference in the MCL-1 levels between control and HL-1 cells treated with INS+Rap (N = 4). However, there was significant difference in the MCL-1 levels between Rap treated and INS+Rap treated HL-1 cells (N = 4, **p<0.02). D) Immunofluorescence staining with anti-MCL-1 antibody and nuclear stain DAPI in HL-1 cells transfected with either (a) Allstars negative siRNA and (b) Allstars negative siRNA and treated with Rap (10 nM), or (c) miR-29 inhibitor cocktail (mirVana miRNA inhibitors for miR-29a, b and c) and treated with Rap (10 nM). Top panel shows DAPI staining. Bottom panel shows anti-MCL-1 antibody staining. Anti-MCL-1 antibody staining was substantially low in Rap treated HL-1 cells transfected with Allstars negative siRNA (b) compared to that in HL-1 cells transfected with Allstars negative siRNA without Rap treatment. Transfection of HL-1 cells with miR-29 inhibitor cocktail reversed Rap-mediated suppression of MCL-1 expression.

Since insulin suppressed miR-29 in HL-1 cardiomyocytes, we posited that insulin would improve expression of MCL-1 in these cells. qRT-PCR analysis showed that treatment with 100 nM insulin increased MCL-1 mRNA levels significantly in HL-1 cardiomyocytes (Fig. 2A). Conversely, treatment with 10 nM Rap suppressed MCL-1 mRNA levels (Fig. 2B). These data suggested that a miR-29-MCL-1 axis, similar to that seen in mouse and human pancreatic β-cells [15] exists in mouse cardiomyocytes and it is regulated by insulin and rapamycin, an mTORC1 inhibitor.

To further characterize the role of mTORC1 substrates S6K1 and translation initiation factor eIF4E binding protein 4E-BP [32], [33] in the suppression of MCL-1, we tested the specific effects of suppressing signaling by each of the mTORC1 substrates on MCL-1 expression in HL-1 cardiomyocytes. S6K1 suppression was achieved by incubation with PF-4708671(2 µM:12 hr), a highly specific S6K1 inhibitor [34]. The 4E-BP in its hypo-phosphorylated form binds to eIF4E and represses translation [33]. mTORC1-mediated phosphorylation of 4E-BP causes dissociation of 4E-BP and activation of eIF4E-induced translation. Therefore, we tested the effect of suppression of eIF4E-induced translation on MCL-1 expression. This was achieved by treatment with 4E1RCat (5 µM:12 hr), a novel suppressor of eIF4E-induced translation [35]. As shown in Fig. 2B, we observed that suppression of S6K1 and eIF4E-induced translation contributed to suppression of MCL-1 expression in HL-1 cardiomyocytes.

These data suggested that mTORC1 regulates MCL-1 in cardiomyocytes. To verify that insulin activated mTORC1 signaling in HL-1 cardiomyocytes, we determined whether insulin treatment (100 nM:12 hr) induced phosphorylation of mTORC1 substrates. Though insulin treatment could induce phosphorylation of S6K1 rapidly, in this study we chose a 12 hr treatment to be consistent with the treatment time used for determining the changes in miR-29 and an MCL-1 mRNA expression in response to insulin in HL-1 cells. The S6K1 is phosphorylated at Thr^389^ by mTOR [32]. The phosphorylated S6K1 (pS6K1) activates Ribosomal protein S6 (pRPS6) via phosphorylation of five evolutionarily conserved residues of RPS6, Ser^235^, Ser^236^, Ser^240^, Ser^244^ and Ser^247^. pRPS6 is implicated in increasing translation and cell size [32], [33]. The mTORC1 also phosphorylates 4E-BP at Thr^37^/Thr^46^. Western blot analysis of the lysates from HL-1 cells treated with insulin confirmed that insulin induced phosphorylation of mTORC1 substrates and their down-stream targets (S6K1 at Thr^389^; RPS6 at Ser^235/236^ and 4E-BP at Thr^37/46^) in these mouse cardiomyocytes (Fig. 2C). Conversely, Rap treatment (10 nM: 12 hr) of HL-1 cells suppressed this effect (Fig. 2C).

Since insulin improved and Rap suppressed MCL-1expression in HL-1 cardiomyocytes, we tested how the exposure of cells to insulin (100 nM: 12 hr) and Rap (10 nM: 12 hr) simultaneously modulates MCL-1 protein levels in these cells. Rap suppressed MCL-1 protein levels significantly (Fig. 2C). However, Rap-mediated suppression of MCL-1 was partly reversed by the presence of insulin (Fig. 2C). This observation suggested that insulin has a protective effect on MCL-1 protein expression.

Since MCL-1 is a target of miR-29 family miRNAs, and Rap treatment increases miR-29 expression, we investigated whether a miR-29 inhibitor cocktail (inhibitors of miR-29a, b and c) would improve MCL-1 expression in Rap treated HL-1 cells. HL-1 cells were transfected with either Allstars negative control siRNA or miR-29 inhibitor cocktail and after 8 hours of transfection subjected to treatment with Rap (10 nM) overnight. Immunofluorescence analysis using anti-MCL-1 antibody showed that Rap treatment substantially suppressed MCL-1 expression in HL-1 cells transfected with Allstars negative control siRNA, but not in HL-1 cells transfected with miR-29 inhibitor cocktail (Fig. 2D). This observation implied that miR-29 family miRNAs regulate MCL-1expression in HL-1 cardiomyocytes.

### General characteristics and the status of cardiac miR-29-MCL-1 axis of 11-week old ZL and ZDF rats

**Figure 3:**
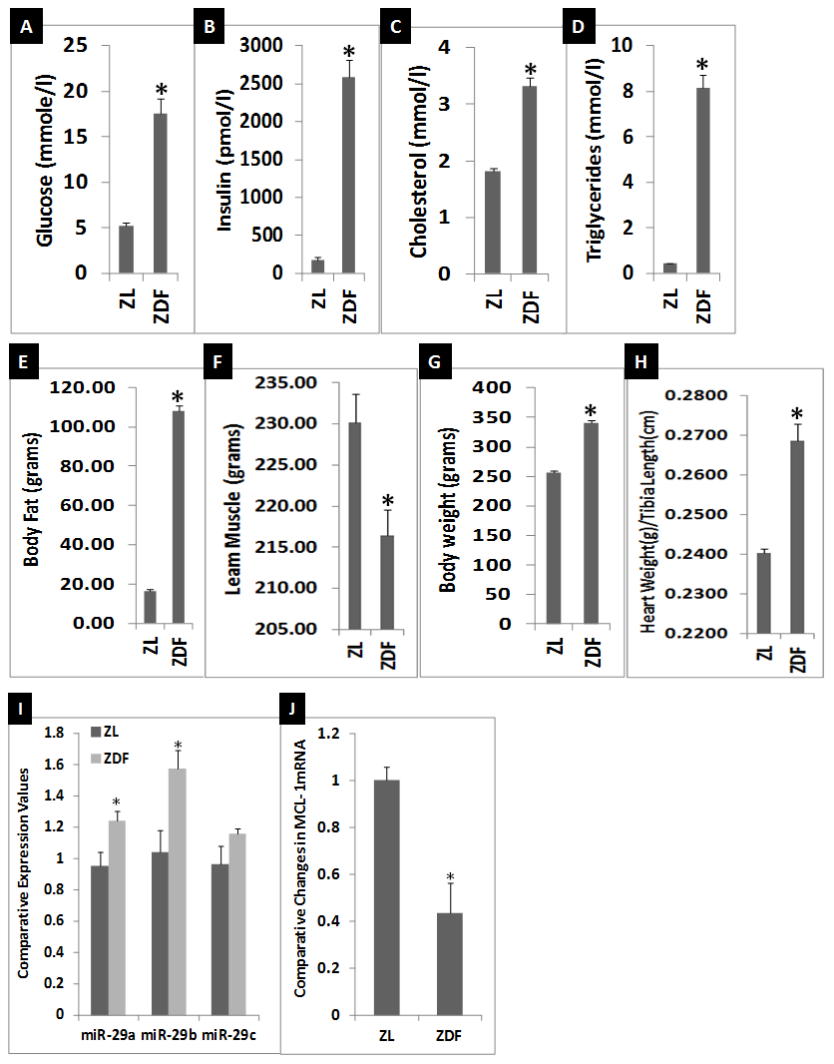
Comparison of the general characteristics and the status of cardiac miR-29-MCL-1 axis of 11-week old ZL and ZDF rats. A-D: Comparison of plasma levels of fasting glucose (A), insulin (B), cholesterol (C) and triglycerides (D). E-I: EchoMRI analysis data on body fat (E) lean muscle mass (F) and body weight (G). H: Heart weight after adjusting to tibia length. I: qRT-PCR analysis data showing the comparative expression levels of miR-29 family miRNAs in myocardium of ZDF rats compared to that in the myocardium of ZL rats. Expression of miR-29a and b were significantly higher in the myocardium of ZDF rat, but miR-29c was not significantly different between ZL and ZDF myocardium. J: qRT-PCR analysis of MCL-1 mRNA levels in myocardium of ZDF rats compared to that in the myocardium of ZL rats. MCL-1 mRNA levels were slightly, but significantly suppressed in the myocardium of ZDF rat compared to ZL rat. n = 7 for plasma analysis and n = 5 for qRT-PCR analysis for ZL and ZDF rats. qRT-PCR for each biological sample was performed in triplicates. Comparative expression levels (RQ values) are expressed relative to ZL myocardium. Values are means ± SEM. *p<0.01 for ZL vs. ZDF.

The ZDF rats are hyperphagic due to a leptin receptor mutation and insulinopenic due to a pancreatic dysfunction [26], [27]. ZDF rats become hyperglycemic by the age of 6 weeks. To verify the general characteristics of ZL and ZDF rats used in this study we determined fasting plasma glucose, insulin, cholesterol and triglycerides at 11-weeks of age. As shown in Fig. 3 (A-D), ZDF rats exhibited significant increases in fasting glucose, insulin, cholesterol and triglycerides at 11-weeks compared to age-matched ZL rats. EchoMRI analysis of the body composition showed that the ZDF rats had substantial increases in total body fat mass and body weight, and a significantly decreased lean muscle mass (Fig. 3E-G). Thus, the ZDF rats used in this study exhibited hyperinsulinemia, hyperglycemia, hypercholesterolemia and muscle loss, characteristic of DM. Heart tissues of 11-week old ZDF rats showed a significant increase in their weight after adjusting to tibia length (3H) indicating cardiac hypertrophy compared to the hearts of age-matched ZL rats. Since ZDF rats had ∼14 fold higher plasma insulin compared to ZL rats (Fig. 3B), at 11-weeks they had compensative hyperinsulinemia. This is consistent with previous reports [26]. qRT-PCR analysis of cardiac miRNA showed that there was a moderate, but statistically significant increase in 29a and b, and a small, non-significant increase in 29c (Fig. 3I). qRT-PCR analysis of cardiac miRNA showed that ZDF rats exhibited about 45% decrease in MCL-1 expression compared to ZL rats (Fig. 3J). These data suggest that cardiac miR-29-MCL-1 axis is mildly dysregulated in 11-week old ZDF rats that suffer from DM.

### Effects of Rap-treatment of ZDF rats on general characteristics and the status of cardiac miR-29-MCL-1 axis

**Figure 4:**
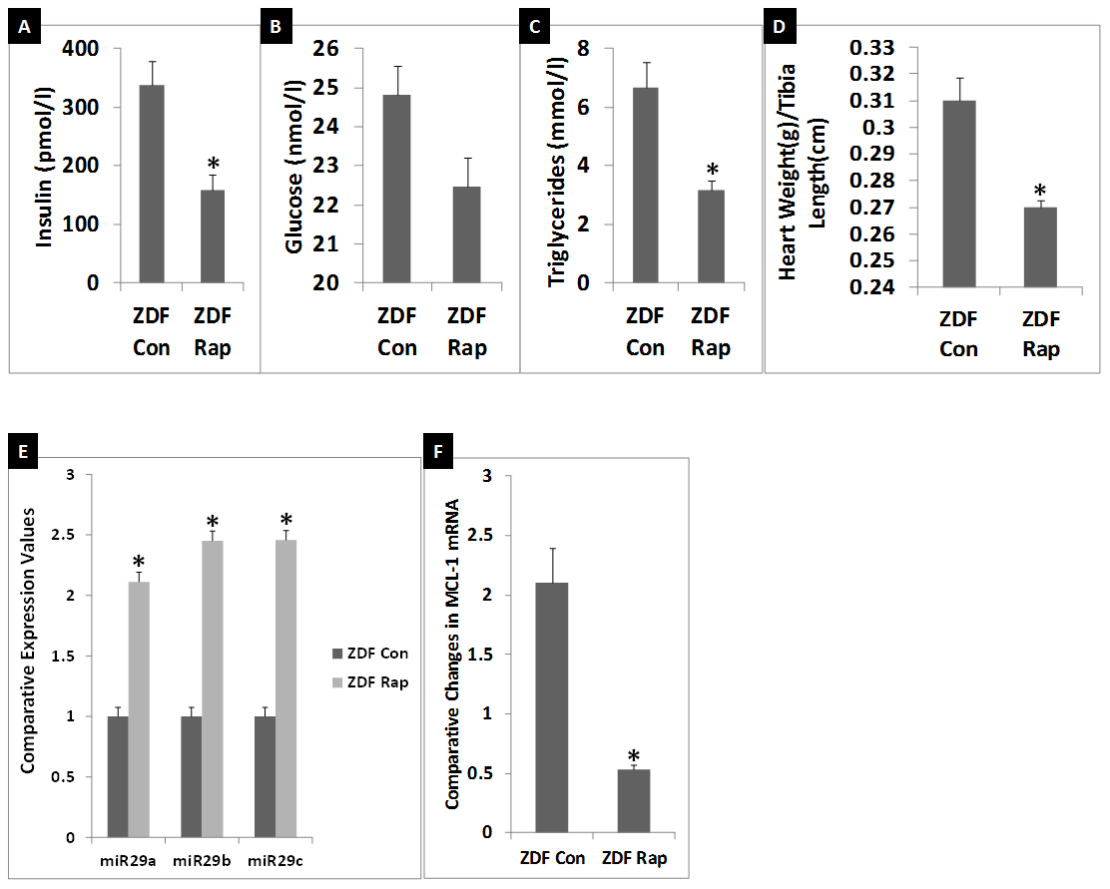
Effects of Rap-treatment of ZDF rats on general characteristics and the status of cardiac miR-29-MCL-1 axis. A-C: Comparison of plasma levels of fasting insulin (A), glucose (B), and triglycerides (C) in control (Con) ZDF rats and Rap treated ZDF rats; (D): Comparison of heart weight after adjusting to tibia length. E: qRT-PCR analysis data showing the comparative expression levels of miR-29family miRNAs in the myocardium of Con ZDF rats compared to that in the myocardium of Rap-treated ZDF rats. Expression of all three miR-29 family miRNAs (a, b and c) were significantly higher (at least 2 fold for each miRNA) in ZDF myocardium. F: qRT-PCR analysis of MCL-1 mRNA levels in myocardium of Con ZDF rats compared to that in the myocardium of Rap-treated ZDF rats. MCL-1 mRNA levels were severely suppressed in the myocardium of Rap-treated ZDF rats (at least five fold). n = 6 for plasma analysis and n = 5 for heart weight analysis and qRT-PCR analysis for Con ZDF and Rap treated ZDF rats. qRT-PCR for each biological sample was performed in triplicates. Comparative expression levels (RQ values) are expressed relative to control ZDF myocardium. Values are means ± SEM. *p<0.01 for control ZDF rat vs. Rap treated ZDF rat.

Since Rap-treatment increased miR-29 levels and suppressed MCL-1 mRNA levels in mouse HL-1 cardiomyocytes, we tested whether Rap-treatment would increase cardiac miR-29 family miRNAs and suppress cardiac MCL-1 mRNA even further in young ZDF rats. Nine-week old male ZDF rats were subjected to Rap treatment (1.2 mg/kg/day) for 6 weeks (until they were 15-weeks old) by implanting Rap pellets subcutaneously. Diabetes in ZDF rats is known to progress from a stage of severe hyperinsulinemia to progressive insulin loss and finally to insulinopenia by the time they are 24 weeks of age [26]. As noted in Fig. 3B, the 11-week old ZDF rats were at a stage of severe hyperinsulinemia (11-week ZDF rat plasma insulin levels: 2587.5±217 pmol/l versus 11-week ZL rat plasma insulin levels: 172.5±48 pmol/l). 15-week old control ZDF rats had a 7-fold reduction in their plasma insulin (Fig. 4A; insulin levels: 337.9±40 pmol/l) compared to 11 week old ZDF rats. Thus 15-week old ZDF rats were at a stage of progressive loss of insulin indicating advancement of DM as described previously (26). Nevertheless, 15-week old ZDF rats were still significantly hyperinsulinemic. Interestingly, in Rap treated ZDF rats, plasma levels of insulin were even more decreased (Fig. 4A: Rap-treated ZDF rat insulin levels: 157.4±27 pmol/l). Therefore, 15-week old Rap-treated ZDF rats had a 2 fold reduction in their fasting insulin levels compared to age-matched control ZDF rats.

Though Rap treatment decreased fasting plasma glucose levels, the Rap-treated ZDF rats still had very high fasting glucose levels (Fig. 4B; fasting glucose in Rap-treated rat: 22.4±0.7 nmole/l) suggesting that they were severely hyperglycemic. Thus Rap-treated ZDF rats were at a more advanced stage of DM compared to age-matched control rats that did not receive Rap since they exhibited severe hyperglycemia with further loss of compensatory hyperinsulinemia. Rap treatment partially decreased plasma triglyceride levels (Fig. 4C) and this is consistent with previous reports. Heart weight (adjusted to tibia length) was further increased in 15-week ZDF rats (Fig. 4D) compared to the heart weight of 11-week ZDF rats (Fig. 3H). Rap treatment reduced heart weight partially in 15-week ZDF rats (Fig. 4D). Suppression of hypertrophy by Rap has been previously reported [36] and the observed reduction of heart weight in 15-week ZDF rat is consistent with this effect.

However, qRT-PCR showed that Rap treated ZDF rats had at least a 2-fold increase in the expression of all miR-29 family members (miR-29a, b and c) (Fig. 4E). qRT-PCR analysis of MCL-1 mRNA levels in Rap-treated ZDF rats showed that there was at least a 4-fold suppression of cardiac MCL-1 mRNA expression in response to Rap treatment (Fig. 4F). These observations suggest that Rap treatment causes severe dysregulation of the miR-29-MCL-1 axis in cardiac tissues of ZDF rat.

### Comparison of cardiac histopathology in 15-week old Rap-treated and control ZDF rats

**Figure 5:**
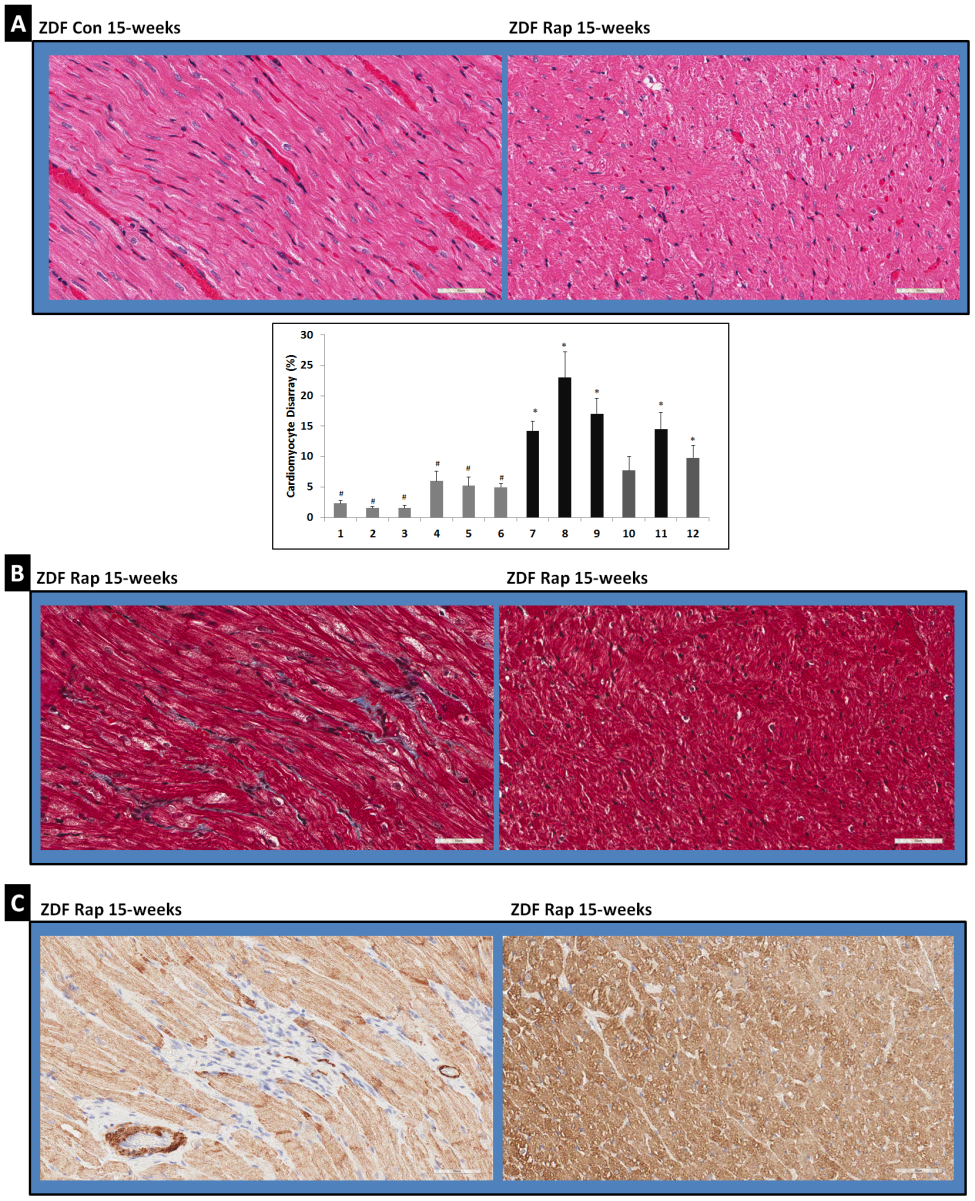
Comparison of cardiac histopathology in 15-week old Rap-treated and control ZDF rats. A) Images of H & E stained sections of necropsy specimens from cardiac right ventricle tissues of 15 week old control rats (ZDF 15Wks) and Rap-treated rats (ZDF Rap15Wks) at different magnifications are shown. Graph shows the percentage of cardiomyofibril disarray in the ventricular sections from control (1–6) and Rap treated (7–12) rats. B) Images of Masson's Trichrome stained sections of necropsy specimens from cardiac right ventricle tissues of two Rap-treated (ZDF Rap15Wks) rats that show cardiomyofibril disarray are shown. In the left panel image some interstitial fibrosis is visible in the region of cardiomyofibril disorganization. However, in the right panel image, interstitial fibrosis was not observed despite evidence of severe cardiomyofibril disarray. C) Images of anti-α-actin antibody stained Rap-treated ZDF rat heart tissues. Fig. 5B and Fig. 5C are showing the same areas of the myocardium of Rap-treated ZDF rats either stained with Masson's Trichrome or by α-actin immunostaining. In the left panel image where there is fibrosis, some loss of α-actin staining is observed. However, in the right panel image, this is not seen. Scale bars: 50 µm.

A recent study reported that there are no significant differences in the histopathology of heart tissues from ZL and ZDF rats in the age group of 12 to 17 weeks [6]. Our histopathology analysis of formalin fixed, paraffin embedded sections of RV tissues stained for H & E and MTS from ZL and ZDF rats used in this study is in agreement with this observation. Though 11-week old ZDF rats had a mild dysregulation of miR-29-MCL-1 axis that resulted in about 45% suppression of MCL-1, no visible differences were observed in the histopathology of the RV tissues between ZL and ZDF rats. However, six weeks of Rap treatment resulted in some visible changes in the histopathology of RV tissue sections of ZDF rats compared to that of age-matched control ZDF rats (Fig. 5). H & E stained sections from necropsy specimens of Rap-treated ZDF rats showed some regions where there was a disorganization of myocardial muscle bundles and damaged tissue and such regions were not observed in the H & E stained sections from necropsy specimens of untreated ZDF rats (Fig. 5A). Quantification of cardiomyocyte disarray in Rap treated and control ZDF rats showed that all Rap treated ZDF rats had an increase in cardiomyocyte disarray. The extent of disarray ranged from moderate to severe. In Masson's Trichrome stained sections from necropsy specimens of Rap-treated ZDF rats, we observed some interstitial fibrosis in one animal that showed severe cardiomyocyte disarray (Fig. 5B, left panel). However, an increase in interstitial fibrosis was not commonly seen in other Rap-treated ZDF rat heart sections (Fig. 5B right panel). Sarcomeric content was visualized by α-actin staining. We observed some loss of α-actin staining in one of the Rap treated ZDF rat heart that showed interstitial fibrosis (Fig. 5C left panel). However, there was no significant difference in the α-actin staining between the heart tissues of Rap treated ZDF rats and control ZDF rats. These observations suggest that the myocardium of Rap-treated ZDF rats that had a further increase in miR-29 a, b and c miRNAs and further suppression of MCL-1 (Fig. 4E and 4F) compared to age-matched control rats, exhibited significant disorganization of myofibril bundles that reflect tissue damage. That was not observed in age-matched control rats. To identify if apoptosis is associated with the disorganization of cardiomyocytes, we performed an indirect TUNEL assay on the formalin fixed, paraffin embedded sections of right ventricle by using ApopTag Fluorescein In Situ Apoptosis Detection Kit S7110 (Millipore). No detectable apoptosis was found in the heart tissues from either control or Rap-treated ZDF rats whereas positive controls for apoptosis exhibited detectable apoptosis by this method (data not shown). This observation is consistent with previous reports that show loss of MCL-1 did not promote apoptosis (16, 17).

## Discussion

Despite extensive epidemiological evidence that suggests DM is an independent predictor of heart disease and heart failure, the exact mechanisms by which DM causes cardiac damage is not clear. An increasing detection of asymptomatic left ventricular dysfunction without overt cardiac disease is noted in diabetic patients as well [37]. However, increased rate of sudden cardiac death is associated with DM and the mechanisms underlying this pathology are unclear [38].

A recent study has reported that in 14-week old ZDF rats there is a significant decrease in RV and LV function compared to age and gender matched ZL rats. Moreover, a similar decrease in RV and LV metabolic rates of glucose utilization measured under hyperinsulinaemic euglycaemic conditions was found in ZDF rats compared to ZL rats [5]. Interestingly, it was also observed that RV myocardium of 12–17 week old ZDF rats did not show any significant structural damage despite the fact that myocardial impulse propagation was impaired in the RV tissues [6]. However, myocardium of about 32 week (8-month) old ZDF rats showed a loss of myocytes [39]. Thus, an intriguing question arises regarding what the mechanisms that contribute to cardiomyocyte loss in DM are, and whether they are just an effect of aging in the background of DM. Many studies have shown that Rap treatment promotes the development of DM [18]–[24]. Therefore, our rationale was that treating ZDF rats with Rap would expedite their DM progression and this would give us an opportunity to compare ZDF rats in the same age group, but exhibiting pathology associated with advanced stages of DM. Such an approach would help to subtract the age factor from the group of factors that contribute to the cardiomyocyte disorganization and loss associated with progression of DM.

Increased expression of diabetic marker miR-29 family miRNAs is seen in rodent models of DM and in young and adult diabetic patients with T1DM or T2DM. We undertook this study to uncover the role of microRNA miR-29 family and its target MCL-1, a pro-survival molecule that is critical for cardiomyocyte survival under stress, in the myocardium damage seen in diabetic heart disease. For this study, we focused on the RV of ZDF rat heart since RV dysfunction from structural and functional perspectives has been described previously in young ZDF rats [5], [6] and therefore the baseline parameters were easy to compare in the context of regulation of the miR-29-MCL-1 axis. Moreover, evaluation of RV myocardium damage serves as a strong indicator of advanced heart disease in young ZDF rats since RV heart failure typically follows LV heart failure.

Our *in vitro* studies on mouse cardiomyocyte HL-1 cells showed that insulin regulates miR-29 family miRNAs (mir-29a, b and c) and improves cardioprotective MCL-1 levels in cardiomyocytes. Conversely, inhibition of mTORC1 signaling resulted in up-regulation of miR-29 expression and suppressed MCL-1 expression in cardiomyocytes. These observations revealed that a miR-29-MCL-1 axis exists in cardiomyocytes. Therefore, we investigated if dysregulation of miR-29-MCL-1 axis is correlated to cardiac damage in DM.

First, we tested the status of cardiac miR-29-MCL-1 axis in the RV tissues of ZDF rats at the age of 11-weeks compared to age-matched ZL rats. Consistent with previous reports [34], [35], ZDF rats showed hyperinsulinemia, hyperglycemia, hyperlipidemia, and increased body weight compared to age-matched lean rats. They also had increased heart weight and reduced lean muscle mass compared to ZL rats. Thus, they were in the hyperinsulinemic stage of DM progression and their hearts were larger despite lean muscle mass reduction. Consistent with a previous report [6], histopathology analysis of ventricular cardiac tissues of ZDF rats did not show any significant pathological changes compared to the cardiac tissues of age-matched ZL rats. We only observed a mild dysregulation of miR-29–MCL-1 axis at this stage and a 45% suppression of MCL-1 mRNA (Figs. 3I and 3J). Hyperglycemia is known to significantly increase miR-29c [9]. However, we did not see a significant increase in miR-29c expression in hyperglycemic 11-week old ZDF myocardium. Since we observed that insulin could suppress miR-29 family miRNAs in cardiomyocyte HL-1 cells, it is conceivable that the lack of significant increase in miR-29c in the myocardium of 11-week old ZDF rats despite severe hyperglycemia could be due to their compensatory hyperinsulinemia (a 14-fold increase in plasma insulin). In brief, the suppression of miR-29 expression by insulin could be a previously unidentified cardioprotective mechanism in hyperglycemia.

Next, we tested the effects of a six-week Rap treatment on 9-week old ZDF rats. At the end of treatment, these rats were 15-weeks old. The dose of Rap (1.2 mg/kg/day) used in this study is within the dose range used to show therapeutic beneficial effects of Rap in other rodent studies to treat cancer or Alzheimer's disease [40]–[42]. We delivered Rap via time-release pellets implanted subcutaneously. Rap treatment partially reduced fasting plasma glucose, triglycerides, insulin levels and heart weight in ZDF rats. A recent report has shown a similar Rap-mediated effect in another DM rodent model, the db/db mouse [43]. In this study, Rap treatment partially reduced glucose levels in db/db mice, however, the mice were still severely hyperglycemic [43]. In our study, Rap-treated ZDF rats were still also severely hyperglycemic, but their insulin levels (157.4±27 pmol/l) were significantly lower than that seen in hyperinsulinemic, hyperglycemic control ZDF rats (337.9±40 pmol/l) and even slightly lower than that seen in healthy normoglycemic 11-week old ZL rats (172.5±48 pmol/l). Thus, Rap-treated ZDF rats served as a model in which the protection from compensatory hyperinsulinemia was significantly reduced compared to the age matched control ZDF rats. This would have led to the up-regulation of all miR-29 family miRNAs in their heart tissues that resulted in the severely dysregulated miR-29-MCL-1 axis in Rap-treated ZDF rats.

Cardiac tissues of 15-week old Rap treated ZDF-rats (Rap treatment from 9-weeks to 15-weeks) displayed a ∼2-fold increase in miR-29 family miRNAs and a 4-fold suppression of MCL-1 mRNA. This severe suppression of the pro-survival MCL-1 mRNA expression correlated with a significant disorganization of myocardial muscle fiber bundles in Rap-treated rats that was not found in either 11 week-old ZDF rats or 15-week old control ZDF rats. Therefore, dysregulation of miR-29-MCL-1 axis caused by loss of insulin and mTORC1 inhibition is a major factor in promoting myocardial damage in DM in ZDF rats.

**Figure 6:**
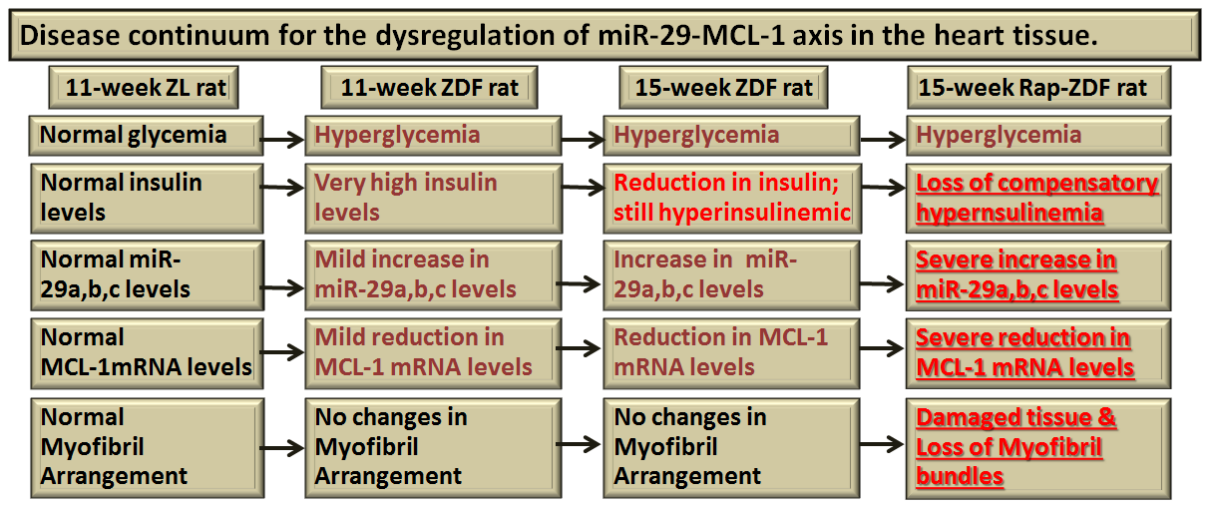
Progressive dysregulation of cardiac miR-29-MCL-1 axis in DM and its correlation with cardiac damage. Summary of our findings is shown. Mild dysregulation of cardiac miR-29-MCL-1 axis in a hyperinsulinemic DM background (ZDF rat) does not show significant cardiac myofibril disorganization or loss. However, suppression of hyperinsulinemia by Rap in the absence of regulation of hyperglycemia as seen in Rap-treated ZDF rat promotes severe dysregulation of cardiac miR-29-MCL-1 axis that leads to disruption and loss of myofibril bundle organization.

Collectively, our data shows the steps in the dysregulation of miR-29-MCL-1 axis in heart tissues during the progression of DM as shown in Fig. 6. Briefly, at the age of 11 weeks, healthy ZL rats show basal level expression of miR-29 and MCL-1 and their myocardium is well organized. 11 weeks old and 15 weeks old ZDF rats have hyperglycemia, but they also have compensatory hyperinsulinemia at different levels. They have mild to moderate dysregulation of miR-29-MCL-1 axis. However, there is no substantial cardiomyofibril disarray. They represent the middle steps in which INS-mediated cardio-protection is still effective. In contrast Rap-treated ZDF rats have very low INS, severe hyperglycemia, and severe dysregulation of miR-29-MCL-1 axis. They have lost the INS-mediated cardioprotection and they exhibit substantial cardiomyofibril disarray (Fig. 6).

## Conclusions

Data presented here shows that insulin down-regulates the expression of diabetic marker miR-29 family miRNAs in mouse cardiomyocytes and preserves the expression of cardioprotective MCL-1. Consistent with this insulin effect, 11-week old hyperinsulinemic ZDF rats only had a mild loss of MCL-1 expression and did not show any damage in myocardium. Therefore, we conclude that regulation of miR-29-MCL-1 axis by insulin is a cardioprotective mechanism and compensatory hyperinsulinemia in conditions of hyperglycemia would regulate miR-29-MCL-1 axis in diabetic heart and prevent significant myocardial damage in young (11- and 15-week) ZDF rats. Conversely, inhibition of mTORC1 signaling by Rap or inhibitors of mTORC1 substrates significantly increased expression of miR-29 family miRNAs and suppressed cardioprotective MCL-1 in mouse cardiomyocytes. Consistent with previous reports, we observed that Rap treatment further suppressed fasting insulin in ZDF rats. Thus Rap-treatment significantly advanced DM in ZDF rats because a two-fold reduction of insulin in conditions of severe hyperglycemia is a step that would expedite DM progression. Moreover, Rap treatment of young hyperinsulinemic ZDF rats caused severe dysregulation of cardiac miR-29-MCL-1 axis and myofibril bundle disorganization indicative of myocardial damage. It is conceivable that such tissue damage in myocardium would increase the vulnerability of diabetic heart to sudden malfunction that results in death.

Rap is known to inhibit pancreatic β-cell proliferation and β-cell adaptation to hyperglycemia. Consistent with this effect of Rap, we observed a significant suppression of fasting plasma insulin levels in Rap-treated ZDF rats. Future studies will investigate how Rap-treatment modulates pancreatic weight, pancreatic insulin content, and β -cell mass and function in ZDF rats.

In this study we used mouse atrial cardiomyocyte HL-1 cells and right ventricular tissues of ZDF rats to investigate how Rap modulates expression of miR-29-MCL-1 axis. Further studies with primary cultures of cardiomyocytes from rat atrium and ventricle are needed to confirm that INS-mediated modulation of miR-29-MCL-1 axis is similar in atrial and ventricular cells. We have shown *in vitro* that a miR-29 inhibitor cocktail could reverse Rap-mediated suppression of MCL-1 protein expression in cardiomyocytes. Further studies are needed to also confirm that in *in vivo* rodent models a miR-29 inhibitor cocktail would improve cardiac MCL-1 protein expression.

Based on the data presented here, we contend that the normal functioning of miR-29-MCL-1 axis is an important cardioprotective mechanism regulated by insulin that exists in female mouse atrial cardiomyocytes and male ZDF rat heart tissue. The extent of loss of this mechanism in response to the progression of DM may determine the extent of cardiac damage as seen in our control versus Rap-treated ZDF rat models (Fig. 6). This observation may have important clinical relevance given the fact that patients with DM are reported to have an increase in miR-29 expression [8], [44]. DM patients are often treated with mTORC1 inhibitors as part of prophylaxis for organ transplant procedures. Though rapamycin has well-established cardioprotective effects, an additional increase in miR-29 family miRNAs due to mTORC1 inhibition in the heart tissues of DM patients can potentially suppress MCL-1 and exacerbate cardiomyocyte disorganization and cardiac damage.
